# Syndromic male subfertility: A network view of genome–phenome associations

**DOI:** 10.1111/andr.13167

**Published:** 2022-03-15

**Authors:** Špela Mikec, Živa Kolenc, Borut Peterlin, Simon Horvat, Neža Pogorevc, Tanja Kunej

**Affiliations:** ^1^ Department of Animal Science, Biotechnical Faculty University of Ljubljana Domžale Slovenia 1230; ^2^ Clinical Institute for Genomic Medicine University Medical Center Ljubljana Šlajmerjeva 4 Ljubljana Slovenia 1000

**Keywords:** genome, male infertility, network medicine, phenome, syndrome, systems biology

## Abstract

**Background:**

Male infertility is a disorder of the reproductive system with a highly complex genetic landscape. In most cases, the reason for male infertility remains unknown; however, the importance of genetic abnormalities in the diagnosis of subfertility/infertility is becoming increasingly recognized. Several syndromes include impaired male fertility in the clinical picture, although a comprehensive analysis of genetic causes of the syndromology perspective of male reproduction is not yet available.

**Objectives:**

(1) To develop a catalog of syndromes and corresponding genes associated with impaired male fertility and (2) to visualize an up‐to‐date genome–phenome network of syndromic male subfertility.

**Materials and methods:**

Published literature was retrieved from the Online Mendelian Inheritance in Man, Orphanet, Human Phenotype Ontology and PubMed databases using keywords “male infertility,” “syndrome,” “gene,” and “case report”; time period from 1980 to September, 2021. Retrieved data were organized as a catalog and complemented with identification numbers of syndromes (MIM ID) and genes (Gene ID). The genome–phenome network and the phenome network were visualized using Cytoscape and Gephi software platforms. Protein–protein interaction analysis was performed using STRING tool.

**Results:**

Retrieved syndromes were presented as (1) a catalog containing 63 syndromes and 93 associated genes, (2) a genome–phenome network including *CHD7* and *WT1* genes and Noonan and Kartagener syndromes, and (3) a phenome network including 63 syndromes, and 25 categories of clinical features.

**Discussion:**

The developed catalog will contribute to the advances and translational impact toward understanding the factors of syndromic male infertility. Visualized networks provide simple, flexible tools for clinicians and researchers to quickly generate hypotheses and gain a deeper understanding of underlying mechanisms affecting male reproduction.

**Conclusion:**

Recognition of the significance of genome–phenome visualization as part of network medicine can help expedite efforts toward unravelling molecular mechanisms and enable advances personal/precision medicine of male reproduction and other complex traits.

## 1 INTRODUCTION

Male infertility is a multifactorial condition that affects approximately 7% of the male population.^1^ Male fertility depends on the process of testes development and associated spermatogenesis. Organized sequential changes in gene expression are required in order to create fully functional testes capable of producing mature spermatozoa.^2^ Additionally, spermatogenesis also requires intact hormonal stimulation from the hypothalamus and pituitary gland.^3^ The Manual for the Standardized Examination, Diagnosis, and Treatment of the Infertile Male, published by the World Health Organization (WHO), states that male fertility can be compromised by congenital or acquired urogenital abnormalities, malignancies, genitourinary tract infections, elevated scrotal temperature (e.g., as a result of varicocele), endocrine disorders, genetic abnormalities and immunological factors.^4^ Most cases of male infertility are presented with low sperm counts and/or poor sperm quality.^5^ Genetic causes are highly heterologous and include chromosomal abnormalities, point mutations in single genes, copy number variations, sequence variants and dysregulation of protein‐coding, miRNA, and mitochondrial DNA genes, polygenic or multifactorial genetic defects, and endocrine disorders of genetic origin.^6^, ^7^ About 4% of infertile men are diagnosed with a genetic cause, and the highest percentage of known genetic factors that accounts for up to 25% of male infertility is azoospermia.^8^, ^9^ However, in about 40% of infertile men, the etiology remains unknown that is also referred to as idiopathic. Some anomalies can be treated surgically to restore male fertility to some degree. For example, undescended testicles are surgically moved into the scrotum. With the development of assisted reproductive techniques (ART), men can reproduce if spermatozoa or earlier stage germ cells can be collected from their testes; however, there is a risk of passing on genetic abnormalities,^10^ especially if they are not molecularly determined beforehand. Therefore, the diagnosis of known and idiopathic male infertility is of clinical importance, moreover, the discovery of novel genetic factors is needed.^1^

A syndrome is characterized as a disorder that has more than one identifying feature or symptom. For some syndromic forms of male subfertility/infertility, the molecular basis is already known; however, for some it remains unidentified. A major reason for this may be that fertility status is not as strongly considered in the recognition and reporting of rare syndromes.^11^ Some of the syndromic forms have reduced fertility as one of the most obvious clinical features, while in the majority, subfertility/infertility is coupled with mental retardation and severe physical deformities. As these individuals are often not concerned with the reproductive health and family planning, they are unaware of their fertility status.^12^ Several genetic syndromes have been reported to be associated with reduced male fertility,^9^, ^13^ yet most of the published studies regarding the syndromology of male subfertility/infertility focused on a handful of syndromes and did not systematically focus on the syndrome aspect. A similar study focusing on syndromes including cryptorchidism in the clinical picture was previously published by Urh et al.^14^ Following this example, a catalog including a wider range of symptoms affecting male fertility needed to be assembled. Even though comprehensive reviews or overviews of validated genetic causes of male infertility have been published,^9^, ^15^ to our knowledge, no such review strictly focused on syndromic infertility to develop a catalog of syndromes and their corresponding genes. Incidence data on 35 of 63 syndromes included in the present study were obtained from the Orphanet reports series.^16^ Twenty‐five syndromes have an estimated incidence of 0.2 to 98/100,000, and in 10 syndromes up to 950 cases were reported. Therefore, the aim of this study was to (1) obtain data on syndromes associated with male subfertility/infertility and, if known, the corresponding genes and present it as a catalog, and (2) visualize a genome–phenome network of syndromic male subfertility/infertility.

## 2 MATERIALS AND METHODS

The initial literature search was performed using the keywords “male infertility,” “syndrome,” and “gene.” In addition, systematic reviews on male infertility were screened to compile a list of syndromes associated with male infertility. The literature was screened in the Online Mendelian Inheritance in Man (OMIM) (https://omim.org),^17^ Orphanet (http://www.orpha.net) (access date: January 2019–September 2021), Human Phenotype Ontology (HPO) (https://hpo.jax.org),^18^ and the National Center for Biotechnology Information (NCBI) PubMed (https://www.ncbi.nlm.nih.gov/pubmed) databases. For the extracted syndromes, a second round of literature screening was performed to obtain additional publications reporting or presenting patients with syndromes affecting male fertility. References found described patients who had been diagnosed with the syndrome and the patient's infertility had been confirmed. Genetic variations associated with the syndrome were extracted from the references that included molecular analysis. In both rounds of the literature search articles dating from January 1980 to September 2021 were reviewed. In addition to OMIM and HPO, Genetics Home Reference (https://ghr.nlm.nih.gov), National Organization for Rare Disorders (https://rarediseases.org), and Genetic and Rare Diseases Information Centre (https://rarediseases.info.nih.gov) databases were used to extract descriptions and basic genetic or chromosomal variations of syndromes, when applicable. Gene names were edited to comply with human genome organisation (HUGO) Gene Nomenclature Committee (http://www.genenames.org).^19^ Terminology was edited in accordance with the proposed initiative of reporting standardization of male infertility.^6^ The network view of syndromes, genes, and symptoms associated with male infertility was created using Cytoscape, a software environment for integrated models of biomolecular interaction networks.^20^ The chromosomal locations of genes were extracted using the Ensembl BioMart data mining tool.^21^, ^22^ The idiogram with marked chromosomal locations of genes was generated using R, version 4.01 with the karyoploteR package.^23^ Protein–protein interaction analysis was performed using Search Tool for the Retrieval of Interacting Genes/Proteins (STRING).^24^ A complete phenome network of syndromes and clinical features, obtained from the HPO and references for male subfertility/infertility was visualized using Gephi.^25^

## 3 RESULTS

**FIGURE 1:**
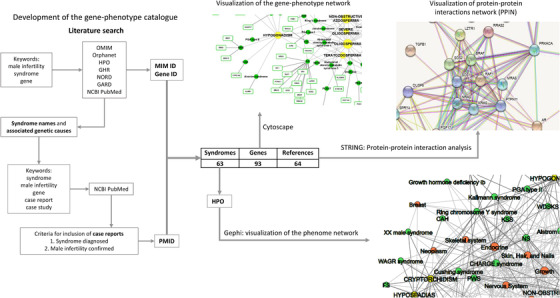
Workflow of the study with main results. OMIM, Online Mendelian Inheritance in Man; HPO, Human Phenotype Ontology; GHR, Genetics Home Reference; NORD, National Organization for Rare Disorders; GARD, Genetic and Rare Diseases Information Center; NCBI PubMed, National Center for Biotechnology Information PubMed, Gene ID: National Center for Biotechnology Information (NCBI) Gene ID, MIM ID, six‐digit identifier in the Online Mendelian Inheritance in Man (OMIM) database; PMID, PubMed identifier number; PPIN, protein–protein interactions network

In the present study, we performed a systematic integration of published data on syndromes with impaired male fertility. The workflow of the study can be divided into the following main phases: catalog development, genome–phenome network visualization, functional enrichment analysis, and phenome network visualization (Figure 1). The retrieved data were organized into a comprehensive catalog, a genome–phenome network, and a phenome network. The catalog contains 63 syndromes and 93 associated genes. Data were extracted from seven databases and 64 published studies. The genome–phenome network contains names of syndromes, associated genes, and eight symptoms leading to male subfertility/infertility.

### 3.1 Development of the catalog

The cataloged genetic variations were divided into two groups: (1) sequence variations in genes and (2) numerical and structural variations in chromosomes. The catalog (Table 1) contains: (1) the names of 63 syndromes alongside 58 identification numbers from the OMIM database (MIM ID) and two from Orphanet (ORPHAcode), (2) the names of 93 associated genes and 93 corresponding gene identification numbers from the NCBI gene database (Gene ID), and (3) 64 NCBI PubMed identification numbers (PMID) of references^26^, ^27^, ^28^, ^29^, ^30^, ^31^, ^32^, ^33^, ^34^, ^35^, ^36^, ^37^, ^38^, ^39^, ^40^, ^41^, ^42^, ^43^, ^44^, ^45^, ^46^, ^47^, ^48^, ^49^, ^50^, ^51^, ^52^, ^53^, ^54^, ^55^, ^56^, ^57^, ^58^, ^59^, ^60^, ^61^, ^62^, ^63^, ^64^, ^65^, ^66^, ^67^, ^68^, ^69^, ^70^, ^71^, ^72^, ^73^, ^74^, ^75^, ^76^, ^77^, ^78^, ^79^, ^80^, ^81^, ^82^, ^83^, ^84^, ^85^, ^86^, ^87^, ^88^, ^89^ reporting syndromes with male infertility. Retrieved references described patients diagnosed with the syndrome and confirmed subfertility/infertility. If the molecular analysis was reported in the reference, the confirmed associations of genetic variations associated with the syndrome were extracted.

**TABLE 1:** Syndromes associated with male subfertility

Syndrome name	MIM ID^a^	Gene symbol^b^	Gene ID^c^	Source of information (database name or reference PMID^d^
Aarskog–Scott syndrome	305400	*FGD1*	2245	OMIM
		/	/	8985497^1^*
Abnormal thyroid hormone metabolism	609698	*SECISBP2*	79048	21084748^2^
Abdominal obesity‐metabolic syndrome 1	605552	/	/	18222914^3^*, 23242914^4^*, 23792341^5^*, 25487258^6^*, 26847036^7^*, 27460460^8^*, 30350486^9^*
Abdominal obesity‐metabolic syndrome 2	605572	/	/	
Abdominal obesity‐metabolic syndrome 3	615812	*DYRK1B*	9149	OMIM
		/	/	18222914^3^*, 23242914^4^*, 23792341^5^*, 25487258^6^*, 26847036^7^*, 27460460^8^*, 30350486^9^*
Abdominal obesity‐metabolic syndrome 4	618620	*CELA2A*	63036	OMIM
		/	/	18222914^3^*, 23242914^4^*, 23792341^5^*, 25487258^6^*, 26847036^7^*, 27460460^8^*, 30350486^9^*
Adrenal hyperplasia due to 3β‐hydroxysteroid dehydrogenase deficiency	201810	*HSD3B2*	3284	OMIM
Alström syndrome	203800	*ALMS1*	7840	OMIM
Androgen insensitivity syndrome	300068	*AR*	367	17970778^10^
Bardet‐Biedl syndrome	209900	*BBS1*	582	OMIM
		*BBS2*	583	
		*BBS10*	79738	
Beckwith–Wiedemann syndrome	130650	*CDKN1C*	1028	OMIM
		*H19‐ICR*	105259599	
		*KCNQ1OT1*	10984	
Bloom syndrome	210900	*BLM*	641	OMIM
Cataract (with non‐obstructive azoospermia)	613887	*TDRD7*	23424	31048812^11^
CHARGE syndrome	214800	*CHD7*	55636	OMIM
		*SEMA3E*	9723	
Complex digit malformation in combination	/	*PDHA2*	5161	29581481^12^
Cone‐rod degeneration with spermatogenic failure	/	*TTLL5*	23093	28173158^13^
Congenital chloride diarrhea	214700	*SLC26A3*	1811	16412765^14^
Congenital adrenal hyperplasia	201910	*CYP21A2*	2600	OMIM
		/	/	22215337^15^, 26666213^16^
Cushing syndrome	615830	*PRKACA*	5566	OMIM
		/	/	195973^17^*, 7650310^18^*, 4835085^19^*
Cystic fibrosis	219700	*FCGR2A*	3616	OMIM
		*CFTR*	1884	
		*TGFB1*	11766	
Deafness‐Infertility Syndrome	611102	*CATSPER2*	117155	17098888^20^ 30629171^21^
		*STRC*	161497	17098888^20^
Deafness with immotile sperm	PS258150, 608653	*CDC14A*	8556	OMIM, 29293958^22^
Denys‐Drash syndrome	194080	*WT1*	7490	OMIM
Frasier syndrome	136680	*WT1*	7490	OMIM
Growth hormone deficiency (type II)	173100	*GH1*	2688	17132747^23^
Kallmann syndrome	308700	ANOS1	3730	OMIM
		*CHD7*	55636	
		*DUSP6*	3072	
		*FEZF1*	22788	
		*FGF17*	3673	
		*FGF8*	2253	
		*FGFR1*	2260	
		*FLRT3*	3762	
		*FSHB*	3964	
		*GNRH1*	4419	
		*GNRHR*	4412	
		*HS6ST1*	5201	
		*IL17RD*	17616	
		*KISS1*	6341	
		*KISS1R*	4510	
		*LHB*	6584	
		*NDNF*	26256	
		*NSMF*	29843	
		*PROK2*	60675	
		*PROKR2*	128674	
		*SEMA3A*	10723	
		*SPRY4*	15533	
		*TAC3*	11521	
		*TACR3*	11528	
		*WDR11*	13831	
Kartagener syndrome	244400	*DNAI1*	27019	OMIM, 11231901^24^, 11713099^25^
Leber congenital amaurosis	611755	*CEP290*	80184	22355252^26^
Congenital generalized lipodystrophy	269700	*BSCL2*	26580	24778225^27^
Muckle‐Wells syndrome	191900	*NLRP3*	114548	22512814^28^
Mulibrey nanism	253250	*TRIM37*	4591	21865362^29^
Noonan syndrome	163950	*BRAF*	673	OMIM
		*KRAS*	3845	
		*LZTR1*	8216	
		*MRAS*	7227	
		*NRAS*	4893	
		*PTPN11*	5781	
		*RAF1*	5894	
		*RIT1*	6016	
		*RRAS2*	17271	
		*SOS1*	6654	
		*SOS2*	6655	
Periventricular nodular heterotopia	300049	*FLNA*	2316	28432720^30^
Persistent Müllerian duct syndrome	261550	*AMH*	268	OMIM
		*AMHR2*	269	
Polycystic kidney disease	173900	*PKD1*	9008	30333007^31^
Polyglandular autoimmune syndrome type I	240300	*AIRE*	326	OMIM
Polyglandular autoimmune syndrome type II	269200	/	/	OMIM
Primary ciliary dyskinesia 9	612444	DNAI2	64446	18950741^32^
Primary ciliary dyskinesia 10	612518	*DNAAF2*	55172	19052621^33^
Primary ciliary dyskinesia 12	612650	*RSPH9*	221421	19200523^34^
Primary ciliary dyskinesia 14	613807	*CCDC39*	339829	21131972^35^, 22693285^36^
Primary ciliary dyskinesia 15	613808	*CCDC40*	55036	22693285^36^, 25619595^37^, 29456554^38^
Primary ciliary dyskinesia 17	614679	*CCDC103*	388389	25877373^39^
Primary ciliary dyskinesia 18	614874	*DNAAF5*	54919	23040496^40^
Primary ciliary dyskinesia 19	614935	*LRRC6*	54562	23122589^41^, 29511670^42^
Primary ciliary dyskinesia 24	615481	*RSPH1*	89765	23993197^43^
Primary ciliary dyskinesia 25	615482	*DNAAF4*	161582	23872636^44^, 28801648^45^
Primary ciliary dyskinesia 32	616481	*RSPH3*	83861	26073779^46^
Primary ciliary dyskinesia 33	616726	*GAS8*	2622	27120127^47^
Primary ciliary dyskinesia 34	617091	*DNAJB13*	374407	27486783^48^
Primary ciliary dyskinesia 36	300991	*DNAAF6*	139212	28041644^49^, 28176794^50^
Tangier disease	205400	ABCA1	19	29198592^51^
Testicular amyloidosis	105200	APOA1	335	15131802^52^, 17507040^53^, 18285420^54^, 24925720^55^, 25565309^56^, 29446975^57^
Woodhouse‐Sakati Syndrome	241080	*DCAF17*	80067	21304230^58^
XX male syndrome	400045	*SRY*	6736	24379036^59^
Young syndrome	279000	/	/	10770909^60^*
**Numerical and structural variations in chromosomes**				
Down syndrome	190685	Trisomy 21		13833938^61^
Jacobs syndrome	ORPHA:8	47, XYY		21671976^62^
Kearns‐Sayre syndrome	530000	various mitochondrial deletions		OMIM
Klinefelter syndrome	/	47, XXY		17415352^63^
Prader‐Willi syndrome	176270	del15q11‐q13		OMIM
Ring chromosome Y syndrome	ORPHA:261529	r(Y)		15214019^64^
WAGR syndrome	194072	Deletions of 11p3		OMIM

Note: /: unknown/not available; *: the reference associated male infertility to the syndrome, but did not conduct a molecular analysis for the cause of infertility in the patient.

^a^ Six‐digit identifier in the Online Mendelian Inheritance in Man (OMIM) database.

^b^ Gene symbol; HUGO Gene Nomenclature Committee (HGNC) (http://www.genenames.org).

^c^ NCBI gene ID.

^d^ PMID: PubMed identifier number.

Fifty‐six of the 63 syndromes are associated with sequence variations in genes, including nine that are associated with multiple genes: Bardet–Biedl syndrome (BBS), Beckwith–Wiedemann syndrome (BWS), CHARGE syndrome, Deafness‐Infertility syndrome (DIS), Kallmann syndrome, Kartagener syndrome, Noonan syndrome, and Persistent Müllerian duct syndrome. Syndromes with the highest number of associated genes are Kallmann (*ANOS1*, *CHD7*, *DUSP6*, *FEZF1*, *FGF17*, *FGF8*, *FGFR1*, *FLRT3*, *FSHB*, *GNRH1*, *GNRHR*, *HS6ST1*, *IL17RD*, *KISS1*, *KISS1R*, *LHB*, *NDNF*, *NSMF*, *PROK2*, *PROKR2*, *SEMA3A*, *SPRY4*, *TAC3*, *TACR3*, *WDR11*) and Noonan syndrome (*PTPN11*, *KRAS*, *SOS1*, *RAF1*, *NRAS*, *BRAF*, *LZTR1*, *SOS2*, and *RIT1*). Seven of the 63 syndromes have numerical/structural variations of chromosomes, of which three have numerical variations (Down syndrome, Jacobs syndrome, and Klinefelter syndrome) and four have structural variations (Kearns–Sayre syndrome, Prader–Willi syndrome, ring chromosome Y syndrome, and WAGR syndrome). For abdominal obesity‐metabolic syndrome 1 (AOMS) and AOMS2, polyglandular autoimmune syndrome type II (PGA II), and Young syndrome, the molecular basis is still unknown.

**FIGURE 2:**
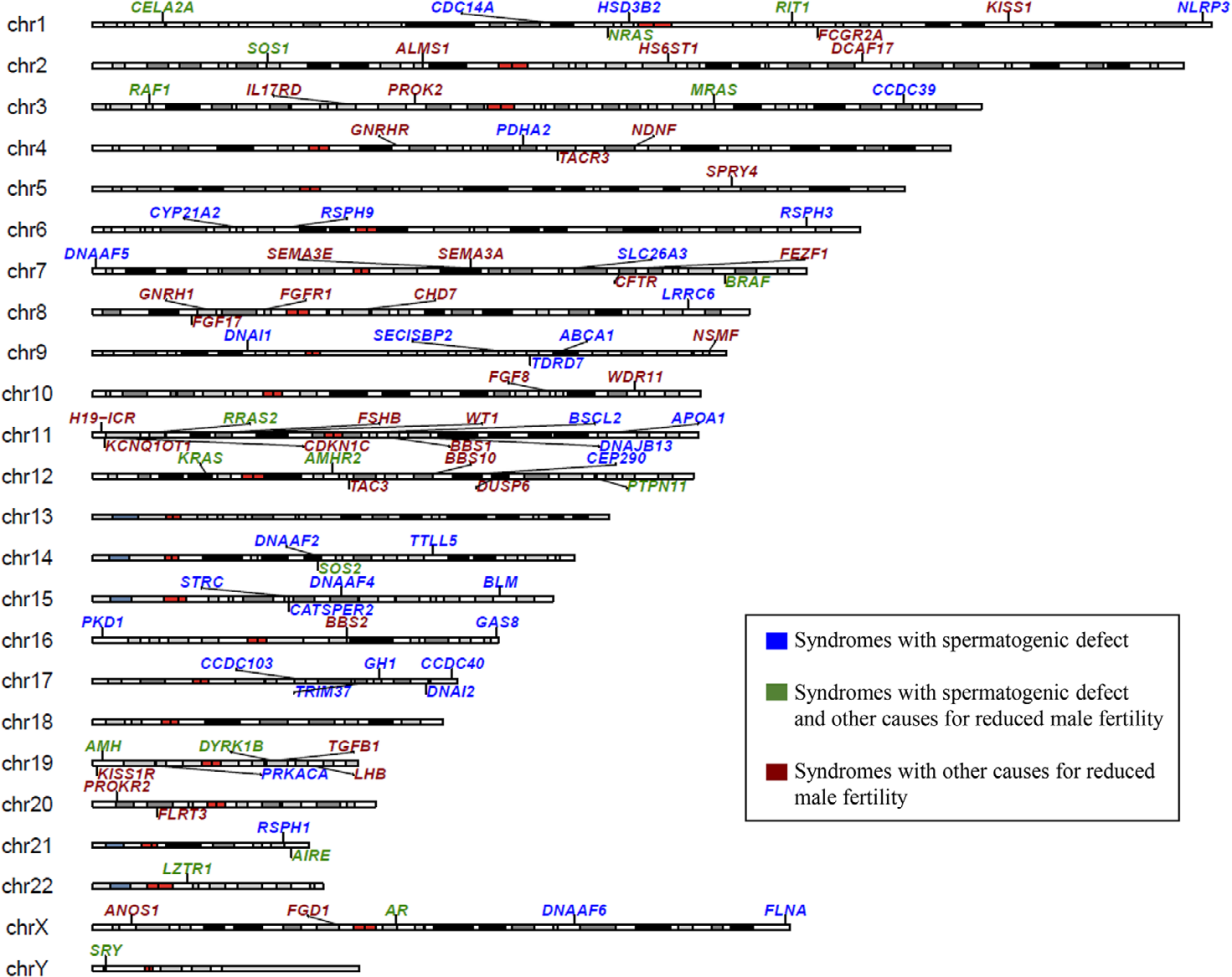
Idiogram depicting genes associated with the cataloged syndromes associated with male subfertility. Legend: Blue, genes are associated with syndromes with reduced male fertility due to spermatogenic defects only. Green, genes are associated with syndromes with reduced male fertility due to both spermatogenic defects and other causes that could lead to male infertility. Red, genes are associated with syndromes in which male subfertility/infertility occurs solely due to obstructive azoospermia, hypogonadism, cryptorchidism, and/or hypospadias

The genomic locations of 93 genes were visualized in an idiogram (Figure 2). Genetic factors were found to be dispersed throughout the genome with no clusters or hotspots observed. However, chromosome 11 was found to contain the largest number of genes (ten) associated with syndromic male subfertility: *H19‐ICR*, *KCNQ1OT1*, and *CDKN1C* are associated with BWS, *FSHB* with Kallmann syndrome, *RRAS2* with Noonan syndrome, *BSCL2* with Congenital generalized lipodystrophy, *DNAJB13* with primary ciliary dystrophy, *APOA1* with testicular amyloidosis, *BBS1* with BBS, and *WT1* with Denys–Drash syndrome and Frasier syndrome. With the exception of chromosomes 13 and 18, there is at least one gene associated with male subfertility located on every other chromosome. The exact cytogenetic locations of all the genes depicted in the idiogram are presented in Table S1.

### 3.2 Visualization of the genome–phenome network

**FIGURE 3:**
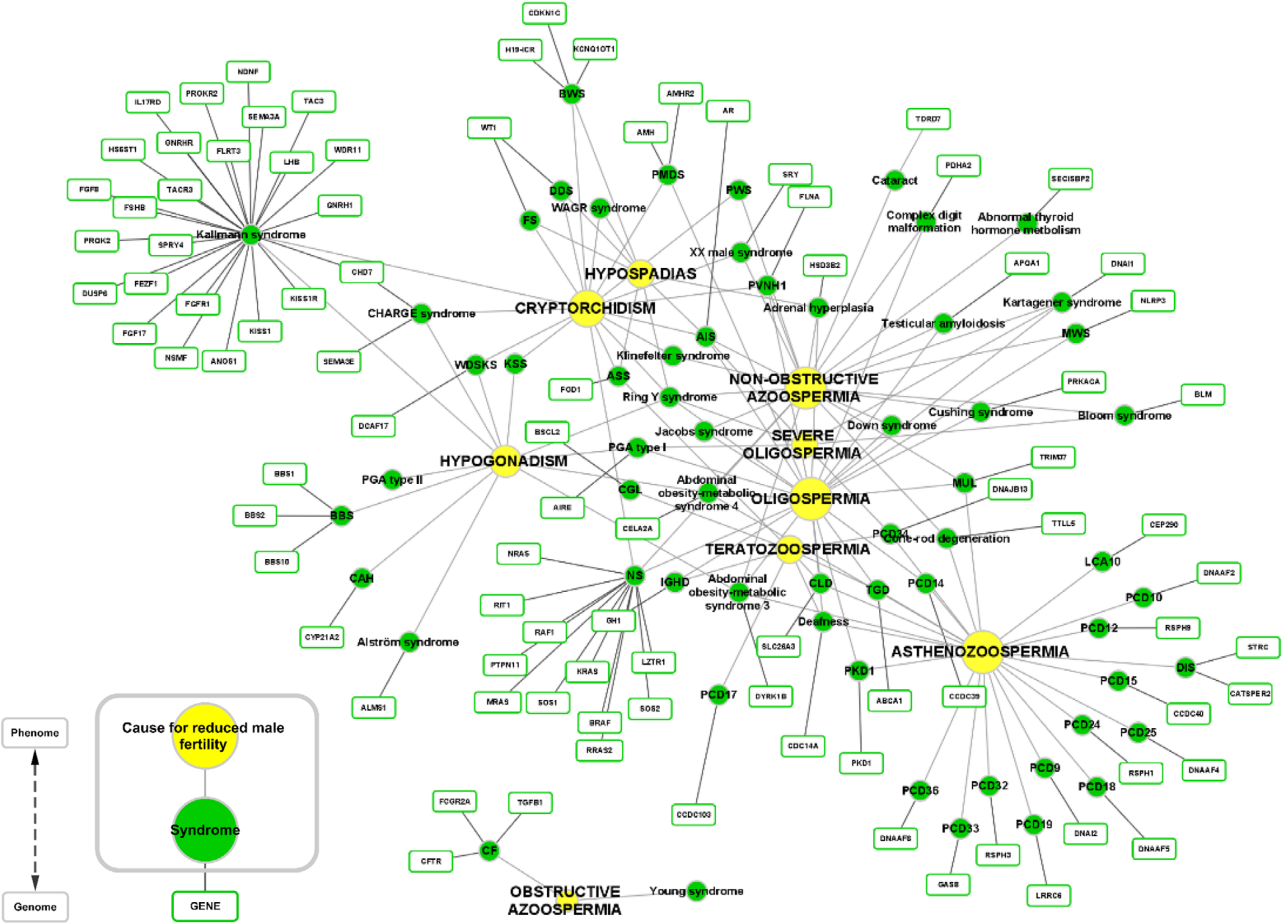
Network of syndromes, symptoms and genes associated with syndromic male subfertility. Legend: Green, syndromes associated with reduced male fertility. Yellow, symptoms depicting causes for reduced male fertility. Green thinly lined rectangles, genes associated with the syndromes. Light gray lines, connect symptoms and syndromes. Dark gray lines, connect syndromes and genes. ASS, Aarskog–Scott syndrome; AOMS, abdominal obesity‐metabolic syndrome; AIS, androgen insensitivity syndrome; BBS, Bardet–Biedl syndrome; BWS, Beckwith–Wiedemann syndrome; CLD, congenital chloride diarrhea; CAH, congenital adrenal hyperplasia; CF, cystic fibrosis; DIS, deafness–infertility syndrome; DDS, Denys–Drash syndrome; FS, Frasier syndrome; IGHD, isolated growth hormone deficiency; LCA, Leber congenital amaurosis; MWS, Muckle–Wells syndrome; MUL, Mulibrey nanism; NS, Noonan syndrome; PVNH, periventricular nodular heterotopia; PMDS, persistent Mullerian duct syndrome; PKD, polycystic kidneys; PGA, polyglandular autoimmune syndrome; PCD, primary ciliary dyskinesia; TGD, Tangier disease; WDSKS, Woodhouse‐Sekati syndrome; KSS, Kearns–Sayre syndrome; PWS, Prader–Willi syndrome

The obtained syndromic forms of male subfertility/infertility, together with the corresponding genes and symptoms affecting male fertility, were visualized in the form of a genome–phenome network (Figure 3). The causes of decreased male fertility were presented as nine clinical features, including five spermatogenic defects: nonobstructive azoospermia (the absence of sperm in seminal fluid), oligospermia (<15 million sperm/ml of seminal fluid), severe oligospermia (<5 million sperm/ml of seminal fluid), asthenozoospermia (reduced sperm motility), and teratozoospermia (abnormal sperm morphology), and four other causes for decreased male fertility: obstructive azoospermia, hypogonadism (defects in the production of sex hormones), hypospadias (abnormal positioning of the urethral opening), and cryptorchidism (undescended testes; the failure of at least one testis and associated structures to descend to their usual position in the scrotum during fetal development).^90^ Two categories of connections were used in the visualization step: (1) between syndromes and symptoms, and (2) between syndromes and genes.

The network consists of 165 nodes, 63 syndromes, nine symptoms, and 93 genes, connected by 216 edges. Among the syndromes, Kallmann and Noonan syndromes have the most connections to other nodes, 27 and 12, respectively. Kallmann syndrome is connected to two nodes with symptoms (cryptorchidism and hypogonadism), and 25 nodes with genes. Noonan syndrome is connected to three nodes with symptoms (nonobstructive azoospermia, oligospermia, and cryptorchidism), and nine nodes with genes. The symptom nodes with the most connections are asthenozoospermia and oligospermia with 23 edges, followed by non‐obstructive azoospermia and cryptorchidism, with 14 and 16 edges, respectively. This indicates that they may be the leading causes for reduced male fertility among our cataloged syndromes. Two nodes representing associated genes were connected to more than one node representing syndromes: the *CHD7* gene, associated with both CHARGE and Kallman syndrome, and the *WT1* gene, associated with Denys–Drash and Frasier syndrome. In the former case, the causes for impaired male fertility are cryptorchidism and hypogonadism, and in the latter case, both include gonadal dysgenesis such as cryptorchidism and hypospadias. Thirty‐six genes are exclusively associated with syndromes in which spermatogenic defects are the only reason for reduced male fertility, mostly asthenozoospermia and azoospermia. Eighteen genes are associated with syndromes that have both spermatogenic defects and other causes for decreased male fertility, mostly cryptorchidism. Thirty‐nine genes have been associated with syndromes in which male fertility is impaired solely due to causes such as cryptorchidism, hypogonadism, and/or hypospadias (Table S1).

The results of the protein–protein interaction analysis for 93 genes associated with syndromic male infertility performed using the STRING tool^24^ is presented in Figure S1. The protein–protein interaction network (PPIN) with connections between 91 nodes and 412 edges shows significantly more interactions than expected (PPI enrichment *p*‐value < 1.0 × 10^−16^), indicating that these proteins are biologically connected as a group. The obtained PPIN now enables the prediction of novel candidate genes for male subfertility based on interacting neighbors. Functional enrichment analysis based on the PPIN revealed KEGG associated pathways: renal cell carcinoma, GnRH signaling pathway, GnRH secretion, chronic myeloid leukemia, and acute myeloid leukemia.

### 3.3 Visualization of the phenome network

**FIGURE 4:**
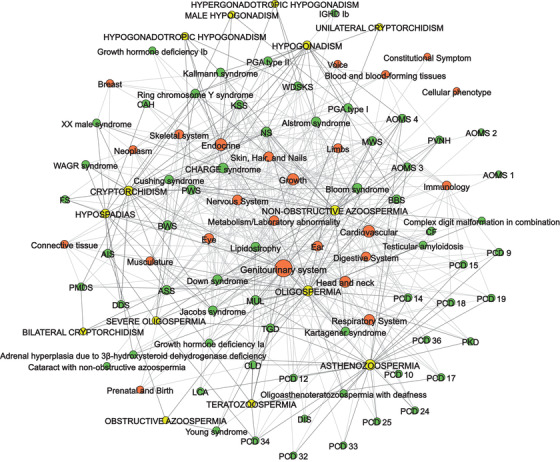
Phenome network of syndromes and associated categories of clinical features. The categories were obtained from the HPO, and information on reduced fertility was obtained from the HPO and case reports from this study. Each category includes many clinical features, for example, BWS is associated with many clinical features that are arranged in the HPO into 15 categories, such as the cardiovascular category including cardiomegaly and cardiomyopathy, and head and neck category, including Dandy–Walker malformation, midface retrusion, coarse facial features, prominent occiput, large fontanelles and prominent metopic ridge. Size of the nodes denotes the number of connections to other nodes. Legend, Green, syndromes, associated with reduced male fertility. Orange, categories of clinical features, associated with investigated syndromes. Yellow, symptoms, depicting causes for reduced male fertility. ASS, Aarskog‐Scott syndrome; AOMS, abdominal obesity‐metabolic syndrome; AIS, androgen insensitivity syndrome; BBS, Bardet–Biedl syndrome; BWS, Beckwith–Wiedemann syndrome; CLD, congenital chloride diarrhea; CAH, congenital adrenal hyperplasia; CF, cystic fibrosis; DIS, deafness–infertility syndrome; DDS, Denys–Drash syndrome; FS, Frasier syndrome; IGHD, isolated growth hormone deficiency; LCA, Leber congenital amaurosis; MWS, Muckle–Wells syndrome; MUL, Mulibrey nanism; NS, Noonan syndrome; PVNH, periventricular nodular heterotopia; PMDS, persistent Mullerian duct syndrome; PKD, polycystic kidneys; PGA, polyglandular autoimmune syndrome; PCD, primary ciliary dyskinesia; TGD, Tangier disease; WDSKS, Woodhouse–Sekati syndrome; KSS, Kearns–Sayre syndrome; PWS, Prader–Willi syndrome

The syndromes investigated in the present study are associated with a range of various clinical features, including male subfertility/infertility, which are further organized into categories. All symptoms and corresponding categories associated with the studied syndromes were obtained from the HPO and presented in Table S2 along with male subfertility/infertility information obtained from the literature inspected in the present study. The 662 clinical features of investigated syndromes are arranged into 25 categories listed along with the number of syndromes associated with the relevant category: genitourinary system (47), cardiovascular (33), growth (30), head and neck (28), endocrine (25), respiratory system (24), nervous system (21), ear (19), eye (19), metabolism/laboratory abnormality (19), skin, hair, and nails (19), digestive system (17), limbs (15), skeletal system (15), immunology (14), musculature (12), neoplasm (10), blood and blood‐forming tissues (8), connective tissue (8), breast (6), prenatal and birth (4), voice (4), cellular phenotype (1), and constitutional symptom (1). A network graph of all syndromes, categories of clinical features, and infertility traits was visualized in Figure 4. The network consists of 102 nodes: 25 categories of clinical features, 14 infertility traits, and 63 syndromes, which are connected by 557 edges.

In addition, a visual presentation (Figure S2) of all syndromes and associated clinical features was created. The network is comprised of 725 nodes and 1234 edges. The nodes represent 63 syndromes and 662 clinical features, including causes for decreased male fertility.

## 4 DISCUSSION AND CONCLUSION

Medical professionals working in andrology need to understand a myriad of genetic abnormalities that alter male fertility so that they can properly counsel couples seeking fertility treatment.^5^, ^91^, ^92^ With the development of ART, males can father children if viable spermatozoa can be found; however, the spermatozoa of infertile males show an increased rate of DNA damage, aneuploidy, and structural chromosomal abnormalities, which poses a risk for passing genetic disorders to their offspring.^92^ Therefore, preimplantation diagnostic procedures should be recommended. For this reason, the pathophysiology of syndromes with impaired male fertility should be further investigated to exclude the possibility of iatrogenically transmitted pathogenic variants. There may be other syndromes with impaired male fertility in the clinical picture; however, we were not able to retrieve them using our search criteria. A major reason for this could be the lack of emphasis on the status of fertility in the detection and reporting of rare syndromes.^11^ Many syndromes, such as Tangier disease and Mulibrey nanism, do not include the word “syndrome” in their names, which adds to the complexity of molecular syndromology field. On the contrary, Sertoli Cell Only (SCO) syndrome is characterized only by a complete or nearly complete absence of germ cells and because it is technically not a syndrome, SCO was not included in the present study. Consequently, there is a great need for more standardized nomenclature guidelines regarding the terminology used in publications for syndromes and clinical symptoms and their relation to ontology terms, such as OMIM, HPO, or DO (Disease Ontology). Furthermore, an estimate of how rare or common the occurrence of syndromic male infertility is within all the causes of male infertility in individual syndromes has yet to be determined.

Despite several advancements of the present study, our analysis has some limitations. (1) Case reports of patients diagnosed with a particular syndrome often present newborns or children, since early diagnosis is imperative for establishing prompt and efficient treatment ensuring a higher level of life quality compared to a later diagnosis. (2) Furthermore, case reports of syndromes, in which the reproductive system is not one of the main affected body parts, do not always specify the fertility status, since other symptoms may have a greater impact on the patient's quality of life. (3) Finally, some case reports do not include a molecular analysis of genetic variations associated with the syndrome analyzed and therefore, the molecular causes of subfertility/infertility in some syndromes still remain to be discovered.

In conclusion, to our knowledge, this study is the first review to focus solely on syndromic male infertility. Although several syndromes were considered, this review needs to be updated with upcoming and potentially overlooked studies. A similar approach could be applied in the future also to other syndromic studies, such as syndromic obesity and other syndromic diseases.

## CONFLICT OF INTEREST

The authors have declared no conflict of interest.

## AUTHOR CONTRIBUTIONS

ŠM and ŽK performed literature screening, curated and interpreted the data, visualized the network using Cytoscape, and drafted the manuscript. ŽK visualized the network using Gephi. NP visualized the idiogram using program R, package karyoplotR. TK and BP conceptualized and coordinated the study and revised the manuscript, furthermore, BP provided scientific advice from the clinical perspective. SH revised the manuscript and interpreted the data. All authors approved the final manuscript.

## Supporting information

Supplementary figure 1. Results of the gene set enrichment analysis and visualization of protein interactions of 93 genes associated with syndromic male infertility using STRING bioinformatics tool.

Supplementary figure S2

Supplementary table 1. Chromosomal locations of 93 genes associated with syndromic male subfertility.

Supplementary table S2
