# Supplementary material for: Syndromic male subfertility: A network view of genome–phenome associations
Source: Andrology. 2022 Mar 15;10(4):720–32. doi: 10.1111/andr.13167 (PMC9314622; doi:10.1111/andr.13167)

**Supplementary figure 1**. Results of the gene set enrichment analysis and visualization of protein interactions of 93 genes associated with syndromic male infertility using STRING bioinformatics tool.


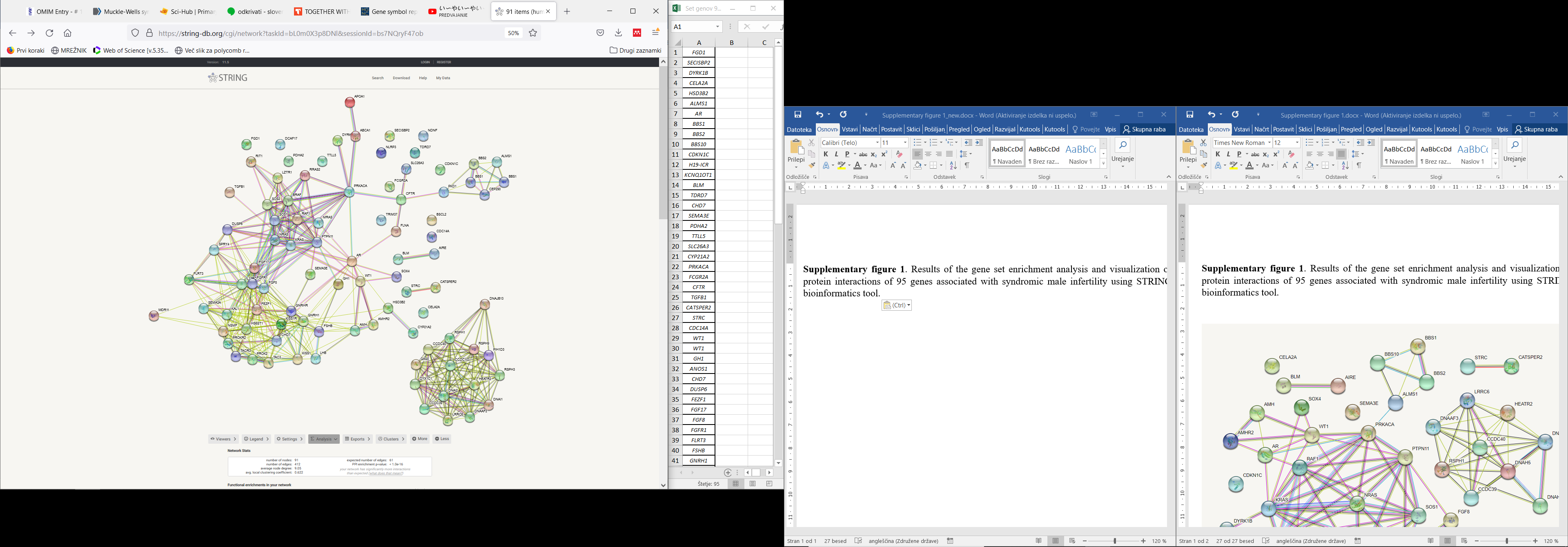


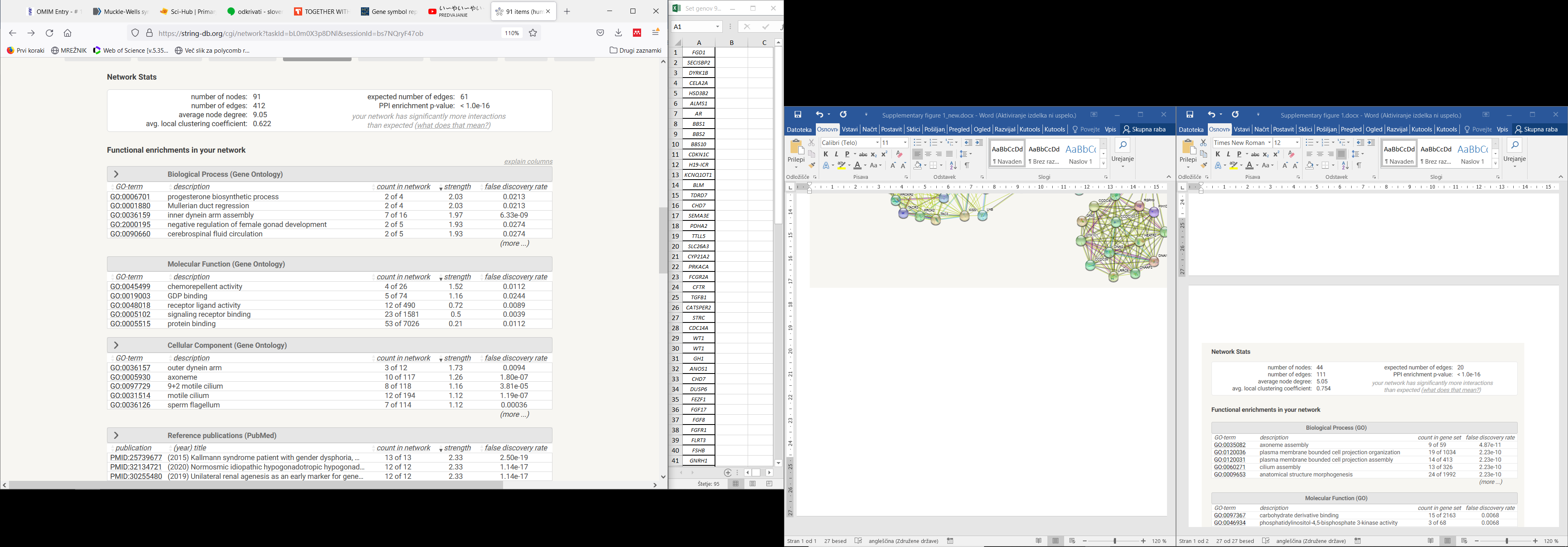


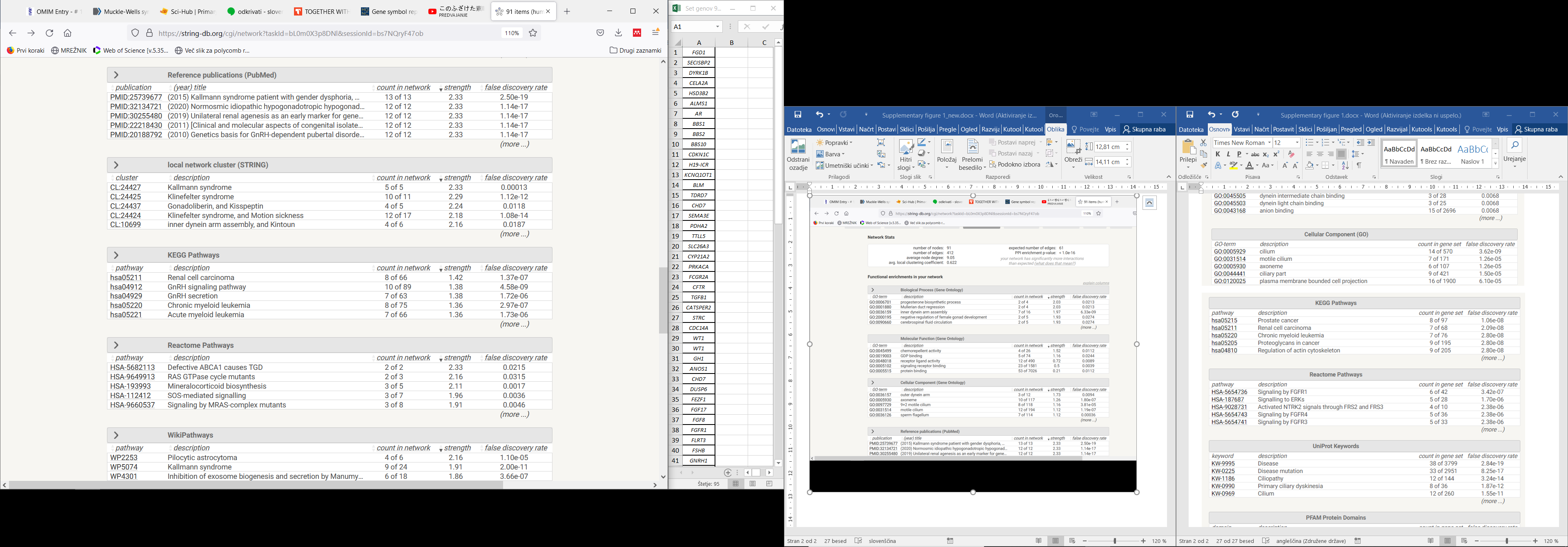


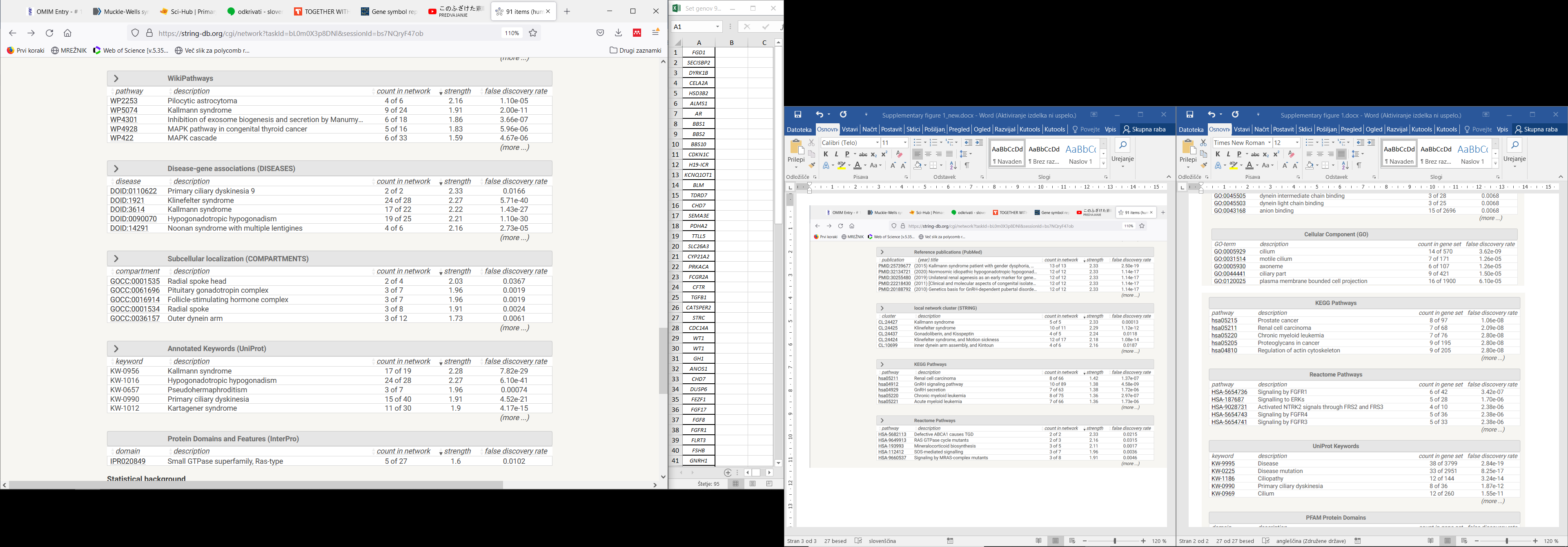

Supplement: Supplementary file 1 — Supplementary figure 1. Results of the gene set enrichment analysis and visualization of protein interactions of 93 genes associated with syndromic male infertility using STRING bioinformatics tool. [file ANDR-10-720-s003.docx]
