# Supplementary figures and images for: Syndromic male subfertility: A network view of genome–phenome associations
Source: Andrology. 2022 Mar 15;10(4):720–32. doi: 10.1111/andr.13167 (PMC9314622; doi:10.1111/andr.13167)

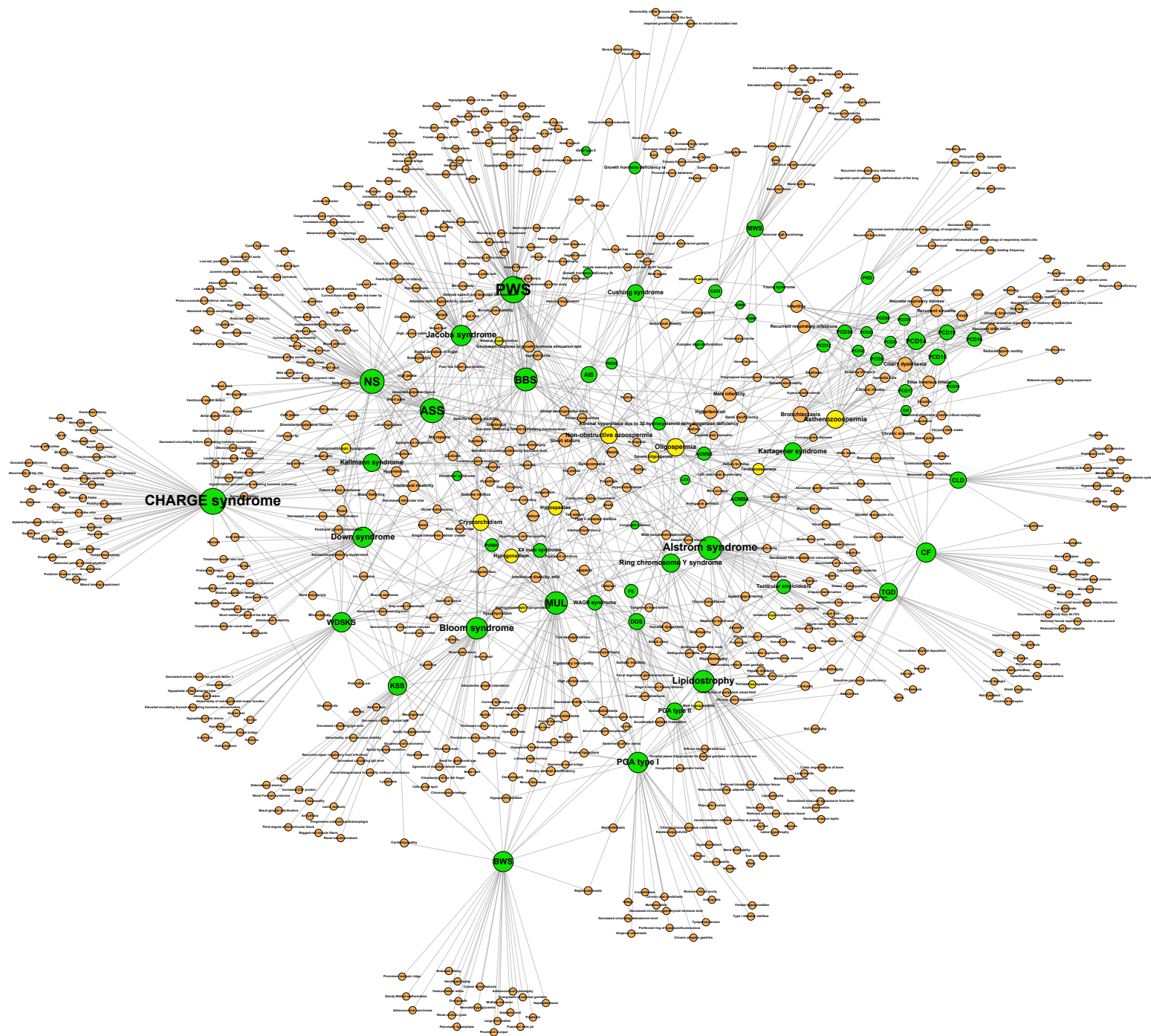

Supplement: Supplementary file 2 — Supplementary figure S2 [file ANDR-10-720-s002.pdf]
