# Supplementary material for: Syndromic male subfertility: A network view of genome–phenome associations
Source: Andrology. 2022 Mar 15;10(4):720–32. doi: 10.1111/andr.13167 (PMC9314622; doi:10.1111/andr.13167)
Supplement: Supplementary file 3 — Supplementary table 1. Chromosomal locations of 93 genes associated with syndromic male subfertility. [file ANDR-10-720-s001.docx]

**Supplementary table 1**. Chromosomal locations of 93 genes associated with syndromic male subfertility.

| **Gene name** | **Cytogenetic location** | **Gene start (bp)** | **Gene end (bp)** |
| --- | --- | --- | --- |
| **Syndromes with spermatogenic defect** | | | |
| *HSD3B2* | 1p12 | [119414931](https://www.ensembl.org/Homo_sapiens/Location/View?db=core;g=ENSG00000203859;r=1:119414931-119423035) | 119423035 |
| *CDC14A* | 1p21.1 | 100345001 | 100520277 |
| *NLRP3* | 1q44 | 247416156 | 247449108 |
| *CCDC39* | 3q26.33 | 180602858 | 180684942 |
| *PDHA2* | 4q22.3 | 95840093 | 95841464 |
| *RSPH9* | 6p21.1 | 43645036 | 43672600 |
| *CYP21A2* | 6p21.33 | 32038327 | 32041644 |
| *RSPH3* | 6q25.3 | 158972871 | 159000202 |
| *DNAAF5* | 7p22.3 | 726699 | 786475 |
| *SLC26A3* | 7q22.3-31.1 | 107765467 | 107803225 |
| *LRRC6* | 8q24.22 | 132570416 | 132675592 |
| *DNAI1* | 9p13.3 | 34457414 | 34520988 |
| *SECISBP2* | 9q22.2 | 89318500 | 89359663 |
| *TDRD7* | 9q22.33 | 97412096 | 97496125 |
| *ABCA1* | 9q31.1 | 104781006 | 104928155 |
| *BSCL2* | 11q12.3 | 62689289 | 62709845 |
| *DNAJB13* | 11q13.4 | 73951026 | 73970366 |
| *APOA1* | 11q23.3 | 116835751 | 116837622 |
| *CEP290* | 12q21.32 | 88049016 | 88142099 |
| *DNAAF2* | 14q21.3 | 49625174 | 49635244 |
| *TTLL5* | 14q24.3 | 75633625 | 75955079 |
| *STRC* | 15q15.3 | 43599563 | 43618800 |
| *CATSPER2* | 15q15.3 | 43628503 | 43668118 |
| *DNAAF4* | 15q21.3 | 55410525 | 55508234 |
| *BLM* | 15q26.1 | 90717346 | 90816166 |
| *PKD1* | 16p13.3 | 2088708 | 2135898 |
| *GAS8* | 16q24.3 | 90019629 | 90044975 |
| *CCDC103* | 17q21.31 | 44899142 | 44905390 |
| *TRIM37* | 17q22 | 58982638 | 59106921 |
| *GH1* | 17q23.3 | 63917200 | 63918839 |
| *DNAI2* | 17q25.1 | 74274234 | 74314884 |
| *CCDC40* | 17q25.3 | 80036632 | 80100613 |
| *PRKACA* | 19p13.12 | 14091688 | 14118084 |
| *RSPH1* | 21q22.3 | 42472486 | 42496246 |
| *DNAAF6* | Xq22.3 | 107206611 | 107244247 |
| *FLNA* | Xq28 | 154348524 | 154374634 |
| **Syndromes with spermatogenic defect and other causes for reduced male fertility** | | | |
| *NRAS* | 1p13.2 | 114704469 | 114716771 |
| *CELA2A* | 1p36.21 | 15456728 | 15472091 |
| *RIT1* | 1q22 | 155897808 | 155911404 |
| *SOS1* | 2p22.1 | 38981549 | 39124345 |
| *RAF1* | 3p25.2 | 12583601 | 12664226 |
| *MRAS* | 3q22.3 | 138347648 | 138405534 |
| *BRAF* | 7q34 | 140719327 | 140924928 |
| *RRAS2* | 11p15.2 | 14277922 | 14364506 |
| *KRAS* | 12p12.1 | 25205246 | 25250936 |
| *AMHR2* | 12q13.13 | 53423855 | 53431672 |
| *PTPN11* | 12q24.13 | 112418351 | 112509918 |
| *SOS2* | 14q21.3 | 50117130 | 50231578 |
| *AMH* | 19p13.3 | 2249309 | 2252073 |
| *DYRK1B* | 19q13.2 | 39825350 | 39834201 |
| *AIRE* | 21q22.3 | 44285838 | 44298648 |
| *LZTR1* | 22q11.21 | 20982269 | 20999032 |
| *AR* | Xq12 | 67544021 | 67730619 |
| *SRY* | Yp11.2 | 2786855 | 2787682 |
| **Syndromes with other causes for reduced male fertility** | | | |
| *FCGR2A* | 1q23.3 | 161505430 | 161524013 |
| *KISS1* | 1q32.1 | 204190341 | 204196491 |
| *ALMS1* | 2p13.1 | 73385758 | 73625166 |
| *HS6ST1* | 2q14.3 | 128236716 | 128318868 |
| *DCAF17* | 2q31.1 | 171434217 | 171485052 |
| *PROK2* | 3p13 | 71771655 | 71785206 |
| *IL17RD* | 3p14.3 | 57089982 | 57170306 |
| *GNRHR* | 4q13.2 | 67737118 | 67754388 |
| *TACR3* | 4q24 | 103586031 | 103719985 |
| *NDNF* | 4q27 | 121035613 | 121073021 |
| *SPRY4* | 5q31.3 | 142310427 | 142326455 |
| *SEMA3E* | 7q21.11 | 83363238 | 83649139 |
| *SEMA3A* | 7q21.11 | 83955777 | 84492724 |
| *CFTR* | 7q31.2 | 117287120 | 117715971 |
| *FEZF1* | 7q31.32 | 122301303 | 122310691 |
| *GNRH1* | 8p21.2 | 25419258 | 25424654 |
| *FGF17* | 8p21.3 | 22042398 | 22048809 |
| *FGFR1* | 8p11.23 | 38400215 | 38468834 |
| *CHD7* | 8q12.2 | 60678740 | 60868028 |
| *NSMF* | 9q34.3 | 137447570 | 137459334 |
| *FGF8* | 10q24.32 | 101770130 | 101780369 |
| *WDR11* | 10q26.12 | 120851305 | 120909524 |
| *WT1* | 11p13 | 32387775 | 32435564 |
| *FSHB* | 11p14.1 | 30231014 | 30235261 |
| *CDKN1C* | 11p15.4 | 2883213 | 2885773 |
| *H19-ICR* | 11p15.5 | 1998745 | 2003509 |
| *KCNQ1OT1* | 11p15.5 | 2608328 | 2699994 |
| *BBS1* | 11q13.2 | 66510606 | 66533613 |
| *BBS10* | 12q21.2 | 76344474 | 76348415 |
| *TAC3* | 12q13.3 | 57010000 | 57028883 |
| *DUSP6* | 12q21.33 | 89347235 | 89352501 |
| *BBS2* | 16q13 | 56466836 | 56520087 |
| *KISS1R* | 19p13.3 | 917287 | 921005 |
| *TGFB1* | 19q13.2 | 41301587 | 41353922 |
| *LHB* | 19q13.33 | 49015980 | 49017091 |
| *FLRT3* | 20p12.1 | 14322985 | 14337614 |
| *PROKR2* | 20p12.3 | 5302040 | 5314369 |
| *FGD1* | Xp11.22 | 54445454 | 54496234 |
| *ANOS1* | Xp22.31 | 8528874 | 8732137 |
